# Management of a Large Quantity of Permanent Gluteal Copolyamide Fillers (Aqualift/Activegel): Literature Review and Algorithm

**DOI:** 10.1093/asjof/ojac051

**Published:** 2022-06-20

**Authors:** Leslie Elahi, Franzisca Ulrich, Wassim Raffoul, Severin Alexander Rossi

**Affiliations:** Department of Plastic, Reconstructive and Hand Surgery, University Hospital of Lausanne (CHUV), Lausanne, Switzerland; Department of Plastic, Reconstructive and Hand Surgery, University Hospital of Lausanne (CHUV), Lausanne, Switzerland; Department of Plastic, Reconstructive and Hand Surgery, University Hospital of Lausanne (CHUV), Lausanne, Switzerland; Department of Plastic, Reconstructive and Hand Surgery, University Hospital of Lausanne (CHUV), Lausanne, Switzerland

## Abstract

Buttock augmentation is a commonly performed aesthetic surgery. Several methods have been described, but only the use of implants or autologous fat is consensually deemed safe and effective. Synthetic fillers in gluteal augmentation have been described despite potential severe long-term complications, both medical and aesthetic. The aim of this study is to report a series of 2 consecutive cases who underwent buttock and hip augmentation with large volumes of permanent copolyamide filler requiring surgical removal due to significant complications. Based on these cases and a review of recent literature, a management algorithm is proposed. The authors conducted a retrospective chart review of 2 consecutive cases of failed copolyamide filler augmentations in the gluteal and inguinal regions. The authors conducted a literature overview using PubMed (National Institutes of Health, Bethesda, MD) and Google Scholar (Google, Mountain View, CA) to include all articles concerning the removal of large quantities of permanent copolyamide fillers. Based on the physical properties of copolyamide, resection of the filler was performed by percutaneous aspiration with liposuction cannulas, with varying infiltration protocols. Both cases showed successful removal of major parts of the filler; however, residual material tended toward migration, requiring a secondary intervention. Hydrated low-pressure aspiration can manage nonintegrated gluteal copolyamide filler but will achieve only partial resection. Literature shows that radical excision is possible, however, with major drawbacks in function and aesthetics. Moreover, in acute inflammation and infection, an open approach should be preferred.

**Level of Evidence: 5:**

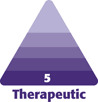

Gluteal augmentation is a commonly performed aesthetic operation. As stated by The American Society of Plastic Surgeons, buttock augmentation has increased by 252% between the years 2000 and 2017. It represents 4.2% of all cosmetic interventions performed in 2019.^1,2^ Several methods have been described^3^: use of buttock implants, augmentation with autologous fat, local flaps and tissue rearrangement (mostly for massive weight loss patients), or the use of fillers. Only autologous fat or the use of buttock implants is approved by the FDA as devices, and they are currently deemed “standard of care.” ^4^ Free liquid silicone injections have been banned since 2006 due to extreme local inflammatory reactions that can lead to mutilating excision surgeries or its potentially life-threatening migration^5,6^ Since then, the market was flooded with new alternative substances with little background and scarce evidence describing their clinical safety. Among these, copolyamide is used mainly in Asia for breast, buttock, or face contouring. Despite articles describing the common complications of this product, particularly in breast augmentation, copolyamide filler is still used for buttock augmentation. Physical properties of copolyamide filler are described in literature^7^ as extremely hydrophilic, liquefying in its hydrated form. Therefore, aspiration of the filler with minimal pressure seems possible in a very aqueous form. We used this method in both cases in order to avoid the extensive scarring and disfiguration of en bloc resection.

The aim of this study is to report 2 consecutive cases of patients who underwent application of large volumes of permanent copolyamide filler requiring surgical removal due to significant negative impact on their health. Based on the experiences made during the treatment of the 2 cases described hereafter, and in association with a review of available literature, we propose an algorithm for copolyamide filler removal.

## METHODS

A retrospective chart review of 2 consecutive cases treated between January 1, 2020, and October 30, 2021, was performed. We included 2 patients with copolyamide complications in this study. Written consent was provided, by which the patients agreed to the use and analysis of their data. The study was conducted in accordance with the Declaration of Helsinki (as revised in 2013). Both were initially treated with low-pressure aspiration after hydration of migrated copolyamide filler. The operations were performed under general anesthesia, in a “day hospital” setting.

Hydration was performed by saline infiltration in a “superwet” ratio (approx. 2:1 infiltration:aspiration) by a MicroAire infiltrator (MicroAire Surgical Instruments, LLC, Charlottesville, VA). Aspiration was performed with a medium bore (3-4 mm) cannula without vacuum first, to allow the majority of liquefied filler to discharge, and then with minimal pressure (<−0.3 bar) on a Vacuson 60 (Nouvag AG, Goldach, Switzerland) or deep manual massages allowing the drainage of the filler without risk of damaging the underlying tissues. Incisions were sutured and stitches removes on postoperative days 10 to 12.

To determine the relevance of our approach, we performed a literature review of studies on the surgical treatment for the removal of large quantities of gluteal copolyamide fillers (main criteria). A review of the PubMed database (National Institutes of Health, Bethesda, MD) and Google Scholar (Google, Mountain View, CA) was realized with the following search inclusion criteria: etiology, complications, similar product polyacrylamide hydrogel (PAAG)/Aquafilling, gluteal/buttock augmentation, epidemiology, and treatment. Excluded from this analysis were articles in which gluteoplasty was performed other than with semi-permanent fillers and article in which other anatomic regions were treated. Due to the paucity of research results, any type of article was accepted in our analysis,x and no tabulations or statistics could be produced. All authors were involved in the research and extraction of the necessary data. Additional articles were considered during the review process.

### Case 1

A 31-year-old female underwent a gluteal augmentation with a 2.2-liter (ie, 22 syringes of 100 mL) copolyamide filler in 2016 in a foreign country. The procedure was performed by a plastic surgeon. She was referred to us after more than a year of disabling pain while sitting, associated with relapsing episodes of erythema and edema usually lasting several days. In addition, she noted a migration and uneven accumulation of the filler in both lower buttocks. She also reported a spontaneous scar rupture of the incisions with filler discharge and healing difficulties several months earlier.

The patient already had several unsuccessful attempts of filler removal before referral to us. On clinical examination, the patient presented unquantifiable subcutaneous palpable nodules in uneven depths of the buttocks, accumulated mainly on the lower and lateral aspects. 

**Figure 1.**
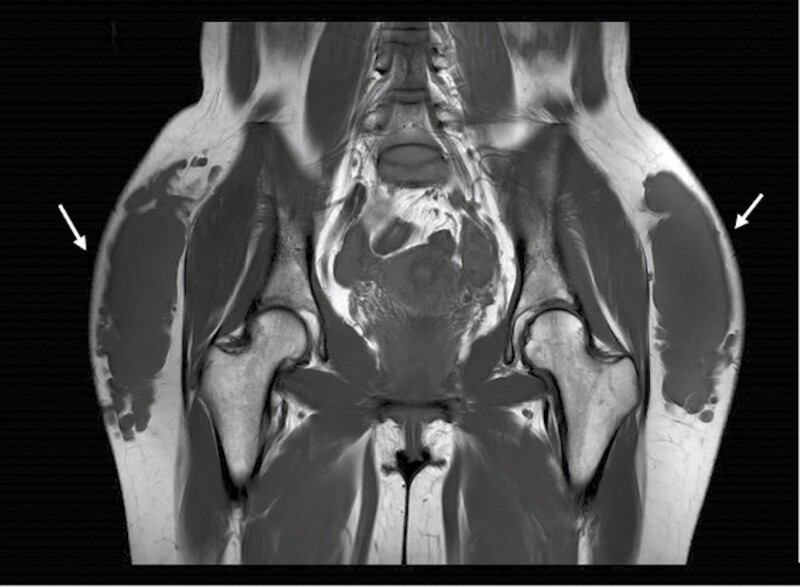
MRI scan of the gluteal region (coronal view): accumulation of poorly defined fillers in different fat layers of both buttocks (white arrows).

**Figure 2.**
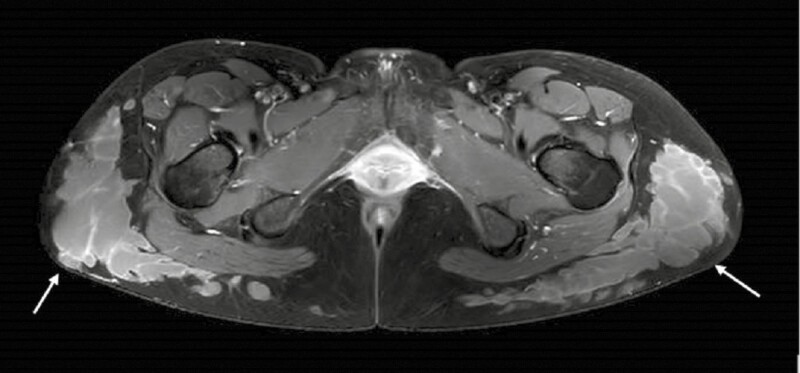
MRI scan of the gluteal region (transversal view): accumulation of poorly defined fillers in different fat layers of both buttocks (white arrows) extending from the gluteal groove to the anterolateral area of the thigh.

MRI confirmed the migration of the filler in superficial and intermediate subcutaneous planes as well as a diffuse inflammatory reaction of the *gluteus maximus* muscle (Figures 1, 2). The patient also presented bilateral reactional inguinal adenopathies and migration of a small quantity of the product to the inguinal folds.

**Figure 3.**
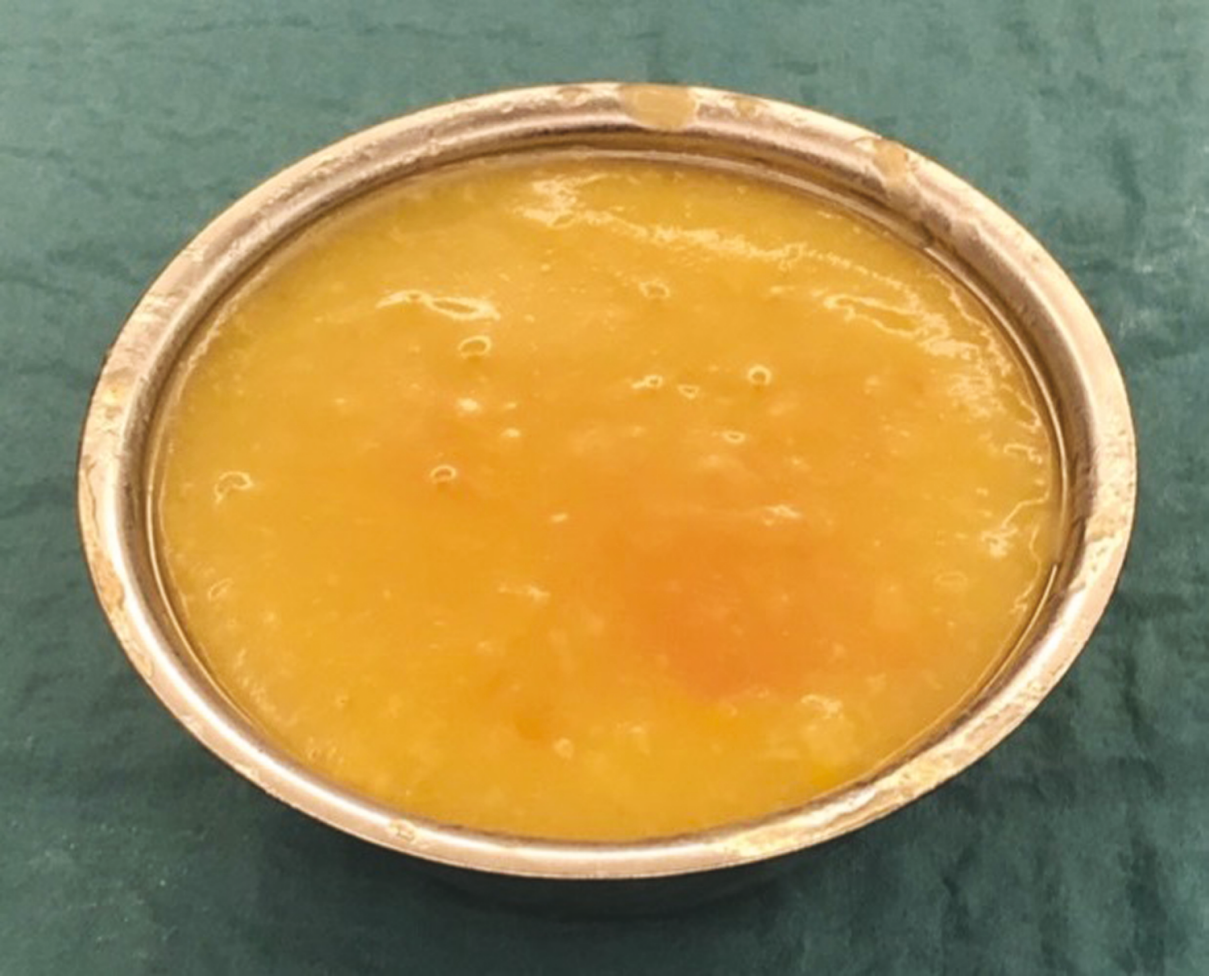
Removal of Aqualift (National Medical Technologies Center Co., Ltd., Ukraine) after 2:1 NaCl infiltration.

**Figure 4.**
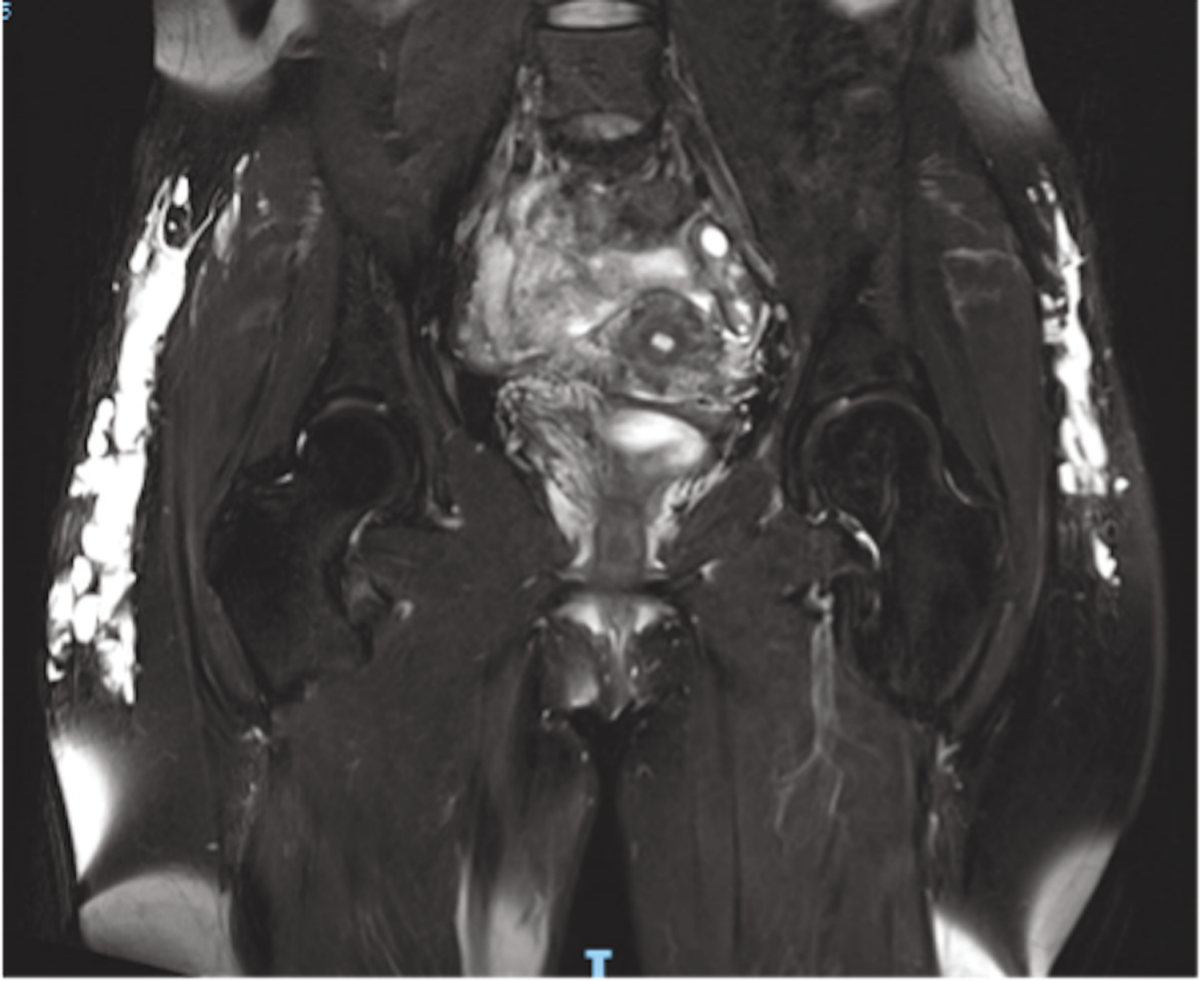
MRI control 3 months after the aspiration: persistence of filler in unquantifiable centimetric vacuoles.

Due to the aesthetic considerations of the patient and absence of inflammation, we chose to resect the filler by hydration and low-pressure aspiration, as described previously. The operation was performed under general anesthesia and day surgery. We used 3.5 liters of infiltration (including 2 liters of Klein solution) for the removal of 4 liters of liquefied filler (Figure 3). Suction was not necessary after infiltration for the removal of the material, and no fat was excised. Manual palpation was sufficient to extract the vast majority of filler, and low-pressure aspiration yielded only about 5% of the total volume. At 1- and 3-month follow-up, the patient did not report any discomfort or pain. However, she complained of persistent palpable nodules on the lateral aspect of the thighs. A follow-up MRI at 3 months showed a decrease in the amount of filler in the buttocks, although a considerable amount was still present in numerous sub-centimetric collections (Figure 4). Unfortunately, she also showed significant migration of filler anteriorly, forming multiple collections, as well as bilateral inflammatory inguinal adenopathy and migration of a small quantity of the product to the inguinal folds. In agreement with the patient, a “wait-and-see” attitude was decided.

### Case 2

**Figure 5.**
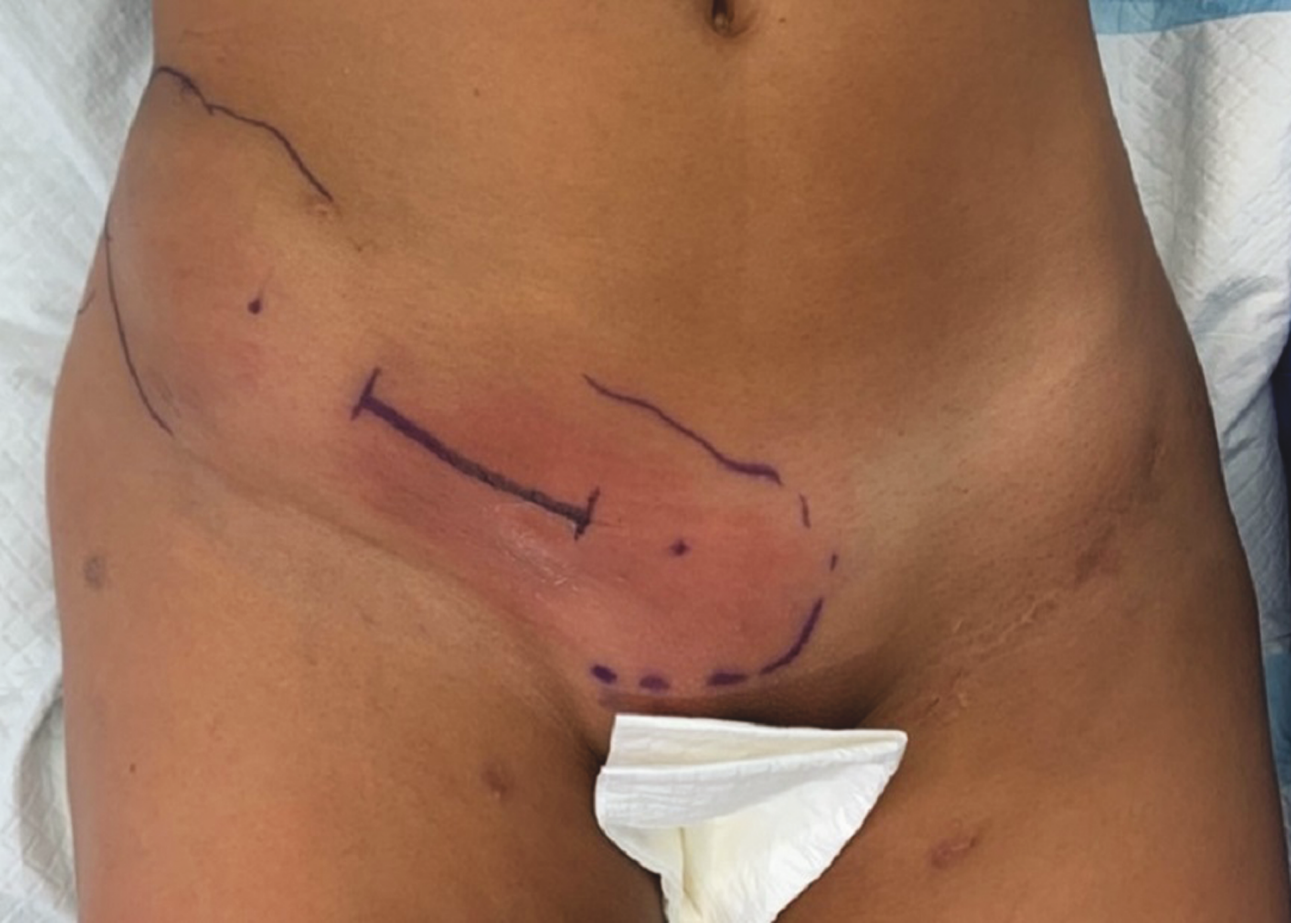
A 24-year-old female patient (patient 2) consulting urgently because of increasing swelling and erythema.

**Figure 6.**
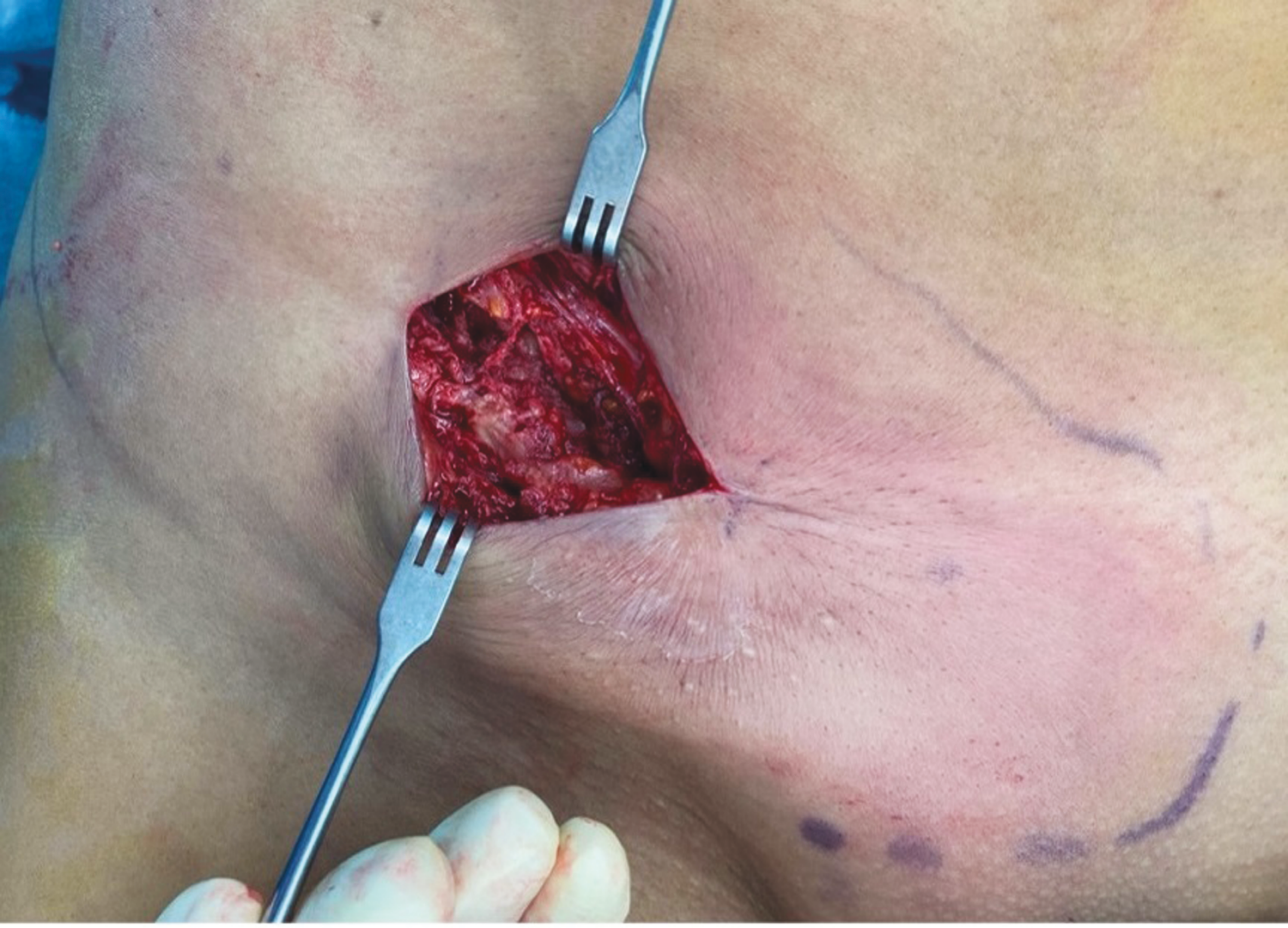
The same 24-year-old female patient featured in Figure 5 at the beginning of the surgery (aspect of the inguinal fold after incision, without dissection). The normal structures are barely distinguishable.

A 24-year-old female was referred to us by general surgeons after multiple gluteal filler infections. The patient had undergone a gluteal and inguinal copolyamide augmentation (unknown volume, but more than 1 liter) in a foreign country in 2016. She was hospitalized and operated on in October 2018 due to multiple subcutaneous abscesses in the inguinal, gluteal, and perineal regions (mostly on the left side) that required multiple incisions. At that time, our colleagues did not suspect filler infection, assuming perineal and sacral abscesses, although localizations were atypical. The patient was subsequently referred to us 1 year after the last abscess incision due to gluteal volume defect following the various debridements. The patient presented retractile and adherent scars with an important depression and atrophy of the subcutaneous tissue. She was also complaining of pain in her right hip, supposably due to a granulomatous reaction of the filler. A first low-pressure aspiration and hydration of the remnant product were performed in June 2020. Several lipofilling sessions with an excellent functional and esthetic result followed. Unfortunately, during fall of 2021, the patient presented with a new inguinal soft tissue phlegmon on the right side. Initially, she presented with an important tumefaction of the hip and inguinal, motivating low-pressure filler aspiration as described previously. Unfortunately, the swelling progressed during the week that followed, with inguinal erythema, tenderness, and fever, requiring incision and drainage (Figures 5, 6). The first intervention with a direct approach was performed, only then could we witness the severe damage of the subcutaneous tissues and lymph nodes. We took samples and closed with a vacuum-assisted closure (VAC) dressing because of the tension of the tissues exerted, allowing us to think about a strategy. VAC dressings were changed every 5 days in a vigilant patient. We waited 2 weeks before taking her back to the operating room. We performed debridement with lymph node sparing under lymphatic indocyanine green fluorescence. Debridement showed necrotic, calcified, infected tissue including the inguinal nodes. Microbiology was never able to isolate a germ, and pathology identified large amounts of amorphous exogen material with gigantocellular reaction, calcinosis, and granulomatosis. The patient was kept on broad-spectrum antibiotics (Piperacillin/Tazobactam) for 5 days before a 5-day treatment of Amoxicillin-Clavulanic acid. Fortunately, the patient did develop only a small seroma, which responded well to manual drainage. We did not do any further imaging because the follow-up was without complication, and the patient did not show any recurrence of phlegmon. She is aware that there is still some material and that a complete excision is not possible without debilitating consequences. We agreed that she will contact us in case of recurrence.

## DISCUSSION

AQUAlift (National Medical Technologies Center Co., Ltd., Ukraine) is a hydrophilic gel composed of 98% sodium chloride solution (0.9%) and 2% copolyamide. It has been renamed as “Activegel” in 2015. This gel is part of the copolyamide filler family, among others Aquafilling (Biomedica, spol, Czech Republic), widely used for breast and lip augmentation and whose complications have been widely reported.^8,9^ AQUAlift/Aquafilling gel is not FDA approved.^10^ There are numerous case reports^9,11-14^ regarding complications related to Aquafilling breast augmentation. The most frequently reported complications include filler migration, late hematomas, skin fistulae, aspecific inflammatory symptoms, breast swelling, and deformity. There are also several large series^15-17^ offering an algorithm of management for breast reconstruction ranging from local filler removal to mastectomy. As stated by Unukovych et al, one of the main problems in the management of these patients is the persistence of filler despite multiple interventions.^17^

There are no established guidelines regarding the methods of removal of large quantities of permanent gluteal filler. The current literature is scarce on the subject. Namgoong et al described 146 cases, among which 6 patients with gluteal augmentation suffered the same type of symptoms/complications that we report.^18^ They chose an open, direct approach combined with the use of a pulsatile jet lavage system to remove the filler with favorable results, although a much larger amount of healthy tissue was sacrificed, leading to significant esthetic impairment. In a letter published in 2016 by the President of the Korean Academic Society of Aesthetic and Reconstructive Breast Surgery,^19^ he expressed concerns about the similarity between copolyamide fillers and polyacrylamide hydrogel (PAAG) that had been withdrawn from the market due to the many reported complications. As a result, the use of copolyamide fillers has been suspended in Korea, except in small volumes in treating wrinkles of the face as well as lips. This similarity was furthermore confirmed by the study “Safety of Copolyamide Filler Injection for Breast Augmentation” by Nomoto et al, showing that these substances share the same chemical composition and complication profile.^14^ Our approach was dictated by the severity of the symptoms, and the mentioned aesthetic compromises accepted by the patients. We are aware that our approach cannot be generalized due to the small number of patients. We seek to share our experience with the aim of raising awareness among colleagues and optimizing treatment protocols. These approaches have been adapted for each situation and should be applied on a case-by-case basis. Because both females were young and did not have severe skin involvement (ulceration or chronic inflammation), they were not willing to undergo en bloc resection. Based on the hydrophilic properties of copolyamide, we assumed that hydration of the filler would allow it to liquefy sufficiently to extract a large quantity. We used a “superwet” infiltration technique (2:1) allowing for manual extraction and low-pressure aspiration. However, it should be noted that infiltration also seems to stimulate filler migration, as shown at radiologic follow-up. Although the patient already showed signs of migration before the procedure, we believe that the amount of infiltration may have influenced the migration of the filler through several mechanisms: hydrodissection of the different subcutaneous layers that could allow the movement of the liquefied filler or even the pressure exerted on the residual liquefied filler postoperatively when the patient sits down. In hindsight, we recommend successive small quantity of infiltrations with the evacuation of the filler each time to prevent this situation. 

**Figure 7.**
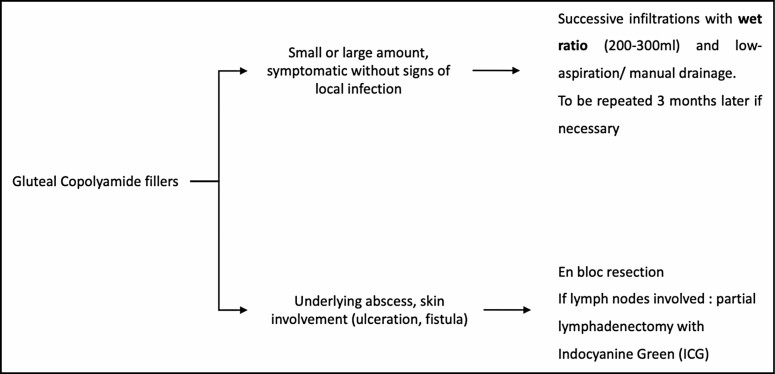
The algorithm for management.

Local inflammation proved to be a challenge to manage in the second patient: filler migration in regional lymph nodes with subsequent infection bore a significant risk of secondary lymphedema in case of radical debridement and lymph node excision. Indocyanine green was of invaluable help to avoid resecting lymphatic tissue while performing a radical debridement of infected tissue and remnant calcified filler. In an acute infection, we do advise an open approach with debridement and rinsing, microbiologic sampling, and antibiotic coverage. Although no bacteria were isolated, symptoms resolved after thorough open rinsing and broad-spectrum antibiotics. The algorithm for management is shown in Figure 7.

## CONCLUSIONS

These 2 cases showed several elements that must be anticipated and emphasized. First, the gel can migrate into the surrounding structures, with or without surgical intervention. Migration can be local (defined as the migration in the same anatomical plane and area) or distant (experience shows a lymphatic migration in regional nodes).

Copolyamide is an active substance that causes granulomatous reactions with potentially severe functional and aesthetic complications that can be triggered years after the first injection. Low-pressure aspiration should be used with caution as a removal method: although esthetically superior to a direct approach, it does not allow for radical removal and can promote local and regional migration. In infectious cases, only a direct approach should be used, as one can remove the maximum amount of filler while preventing migration. The process of removing the filler requires several consecutive interventions, especially if it is associated with a local infection where several washings and debridements are necessary to get rid of all the incriminated material.

Finally, copolyamide fillers can be associated with a multitude of nonspecific symptoms (edema, chronic fatigue, and headaches) in the long term that improve or resolve once the product is removed. Due to the increasing democratization of cosmetic procedures and aggressive advertising, patients seem to be choosing supposedly inexpensive cosmetic procedures with substances that do not have sufficient evidence regarding long-term safety. The amount of complications after such treatments is likely to increase over time, and more studies are required to assess complication profiles of injectable substances both quantitatively and qualitatively, in order to raise patient and professional awareness.
